# Odontogenic Myxoma: Follow-Up of 13 cases after conservative surgical treatment and review of the literature

**DOI:** 10.4317/jced.58080

**Published:** 2021-07-01

**Authors:** Hélder-Domiciano-Dantas Martins, Etiana-Lopes Vieira, André-Luiz-Marinho-Falcão Gondim, Haroldo-Abuana Osório-Júnior, José-Sandro-Pereira da Silva, Éricka-Janine-Dantas da Silveira

**Affiliations:** 1 DDS, MSc Student, Department of Oral Pathology, Federal University of Rio Grande do Norte, Natal, Rio Grande do Norte, Brazil; 2 DDS, Department of Dentistry, Federal University of Rio Grande do Norte, Natal, Rio Grande do Norte, Brazil; 3 DDS, Department of Oral and Maxillofacial Surgery, Federal University of Rio Grande do Norte, Natal, Rio Grande do Norte, Brazil; 4 DDS, MSc, PhD, Professor, Department of Oral and Maxillofacial Surgery, Federal University of Rio Grande do Norte, Natal, Rio Grande do Norte, Brazil

## Abstract

**Background:**

We aim to report a serie of odontogenic myxoma over a 40-year period.

**Material and Methods:**

We conducted a retrospective and sectional review of OM cases. The clinical, radiographic and treatment data were collected from clinical records included cases whose medical records contained clinical, radiographic, histopathological, follow-up data of at least six months.

**Results:**

There was a mild preference for the male with a mean age of 22.8 years. Seventy-seven percent of the lesions occurred in the posterior region of the mandible, presenting a multilocular radiolucent aspect (54%) with one presenting symptomatology. Conservative treatment was performed in all patients initially and recurrence was observed in two cases.

**Conclusions:**

Odontogenic myxoma has a well-defined clinical profile and the choice of treatment should consider aspects such as patient’s age, lesion size, and location.

** Key words:**Myxoma, odontogenic tumors, diagnosis, treatment.

## Introduction

The jawbones can be affected by various types of injuries from different origins. Among these, even benign odontogenic tumors, in some cases, may present locally aggressive and infiltrative behavior (1,2). Since its original description in 1947 by Thoma e Goldman (3), the nature of the OM has been a matter of controversy. It is defined as a relatively rare benign odontogenic neoplasm that represents about 3 to 6% of odontogenic tumors (4). Occurs mainly in young adults (second and third decades of life), with no preference for sex (5,6). Its origin is believed to be related to changes in the ectomesenchyme of a developing tooth or undifferentiated mesenchymal cells of the periodontal ligament.

It ifs clinically presented as a slow-growing lesion, usually asymptomatic, but locally aggressive, as it can cause bone resorption, soft tissue infiltration, and dental alterations (7). The treatment of OM ranges from conservative enucleation and curettage to block resection and hemimandibulectomy (2). Segmental resection is the most accomplished treatment followed by enucleation associated with surgical curettage and partial resection with all presenting similar recurrence rates. 

OM is associated with a possible aggressive behavior and high recurrence rates with a mean of 25%, and understanding of how modality of treatment influences the outcome is still under scrutiny because there is no properly standardized studies (8-10). There are only few reports in the literature about this lesion without bring follow up period. Based on this, we retrospectively analyze the clinical and radiological features of odontogenic myxomas diagnosed over a 40-year period and compare results with the current literature.

## Material and Methods

This study followed the Declaration of Helsinki and resolution 466/12 on medical protocol and ethics and the regional Ethical Review Board of Federal University of Rio Grande do Norte approved the study, according to Opinion No. 1,177,339.

A retrospective and cross-sectional study of cases of OM diagnosed at the UFRN Department of Oral Pathology and followed at the UFRN Department of Oral and Maxillofacial Surgery, Natal-RN, Brazil during the period from January 1985 to February 2019.

The sample included cases whose medical records contained clinical, radiographic, histopathological, follow-up data of at least six months and patients who agreed to participate in the study and attended for clinical and radiographic follow-up. All patients underwent panoramic radiographs for diagnosis, treatment planning, and follow-up.

## Results

During the research period, 23 cases of patients with OM were diagnosed, but only 13 patients attended for follow-up (Table 1). Of the 23 cases, males were the most affected in 53.84% of cases, with ages ranging from 14 to 42 years, and the average age of 20.38 years.

**Table 1:**
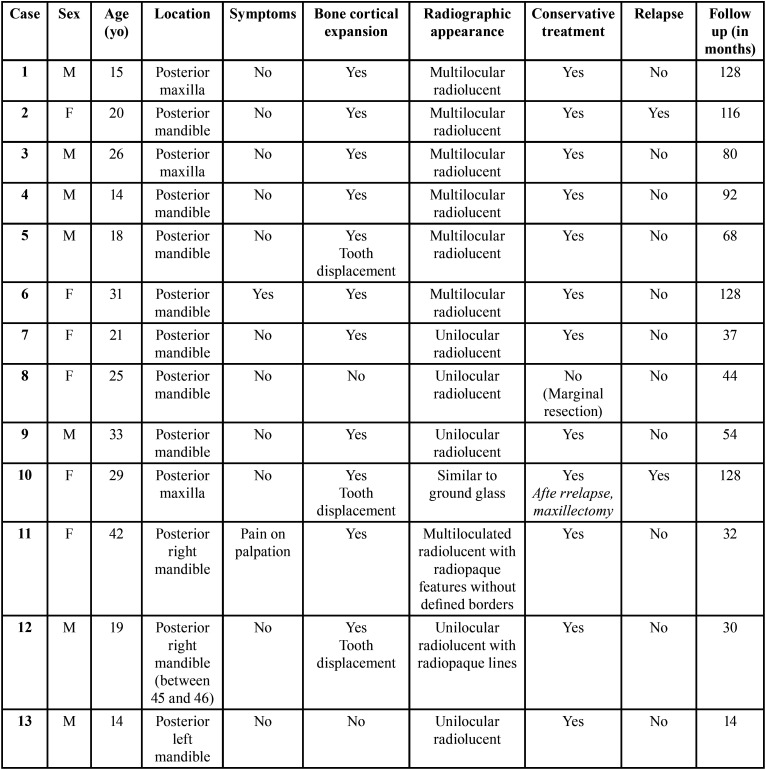
Clinical, radiographic data, treatment and follow-up in 13 cases of OM.

The lesions were in the posterior mandible (77%) and posterior maxilla (23%). Only one case had painful symptoms, and 77% of cases had bone cortical expansion. Three cases exhibited tooth displacement, and most lesions presented multilocular radiolucent appearance (54%).

**Figure 1:**
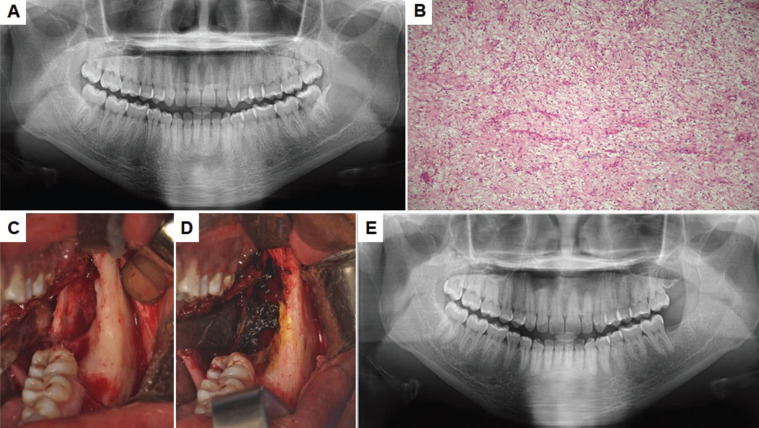
A) Initial aspect: Multilocular radiolucent imaging in left region of mandible B) Histopathological aspect of the lesion after incisional biopsy showing myxoid connective tissue stroma containing few collagen fibers, in which spindle, rounded and stellate cells were observed C) Transurgical aspect after enucleation and peripheral ostectomy D) Use of Carnoy’s solution for 5 minutes in direct bone contact E) Follow up after 29 months with no signs of relapse.

12 patients who came for follow-up were initially treated conservatively, with curettage removal followed by modified Carnoy’s solution and peripheral ostectomy. Only one patient received an aggressive treatment initially. The Figure 1 (A-E) shows a case of OM treated by curettage and Carnoy’s solution. There was a recurrence in two patients (15.38%).

Microscopically, all cases revealed a classic pattern with a predominance of stellate and spindle cells in loose myxoid stroma with dense collagen fibrils and delicate fibers. Binucleated cells and mitotic Figures were rare identified. Vascularity was minimal and no evidence of encapsulation were detected.

## Discussion

Selecting the modality of treatment for OM is a controversial topic in the literature due to its benign nature but high recurrence rates, often attributed to incomplete lesion removal due to the gelatinous and friable appearance of the tumor (2,8).

This lesion is an uncommon neoplasm of the maxillary bones and represents between 2.3 and 3.4% of all odontogenic tumors (11-13), which could be evidenced in the present study with only 23 cases out of a total of 16,000 cases diagnosed in the maxillary bone over 40 years. The average age of the patients was 20.38 years and agreed with the information available in other studies, which indicate that the age group most affected by OM is in the second and third decade of life (5,13). Odontogenic myxoma is rare in patients under ten and over 50 years of age, and no cases were found in these age groups in the present study.

OM is a slow-growing and usually asymptomatic tumor (4,6) and although there is controversy regarding the most frequent location of the OM (14,15), a higher prevalence was observed in the posterior mandible (77%) and maxilla (23%) regions, with no cases in the anterior regions. In addition, only one case presented painful symptoms, 80% of the patients showed cortical bone expansion, and three cases exhibited tooth dislocation.

The radiographic characteristics of the OM may range from small unilocular radiolucent imaging to extensive multilocular lesions with well-defined or diffuse margins and a thin trabecular bone within their structure resembling “honeycombs” or “soap bubbles” (2,9). The accurate radiographic appearance is essential in order to arrive at a correct diagnosis and proper surgical planning, and cone-beam computed tomography is useful in demonstrating the internal structure of the OM (16,17). In the present study, most lesions showed radiographic images (54%) of multilocular radiolucent aspect, without root resorption on panoramic radiographs.

The decision on the therapeutic strategy of the minimally invasive excision biopsies to block resections seems to be influenced by multiple factors such as the size and clinical behavior of the lesion, location, patient age, and surgeon experience (6,17). For young patients, conservative procedures are suggested due to complications related to more aggressive treatment (17). However, even in adults, more radical approaches with broad safety margins do not mean lower recurrence rates (6). Conservative treatment is proposed as a first option based on enucleation followed by bone curettage for small lesions (5,15).

Also, Carnoy’s solution without chloroform can be applied because it is a cauterizing agent that causes rapid local fixation, used directly on the bone bed after enucleation of the lesion to eliminate residual tissue, decrease the likelihood of recurrence and is not carcinogenic (18,19). This approach has the advantage of preserving hard and soft tissue while maintaining the masticatory function. All patients in this series were treated by enucleation associated with peripheral ostectomy, except for case 8, and use of Carnoy’s solution for 5 minutes in contact with the bone bed.

Currently, new evidence suggests that more conservative approaches result in accepTable recurrence rates with lower patient morbidity in a long-term follow-up (8). However, there is a lack of information about the actual follow-up of patients with OM after conservative surgery. The understanding of how modality of treatment influences the outcome is still under scrutiny (8,9). Interestingly, this study reports successful cases in conservative approach in the follow-up of up to 10 years with only two recurrences, showing that enucleation combined with modified Carnoy’s solution can be adopted as a first-line treatment modality to OM.

The follow-up of patients with OMs should be prolonged, depending on the likelihood of recurrence, especially in the first two years. Recurrence rates decreased from 24% to 8.3% among patients who were conservatively treated with follow-up longer than 60 months (8), which was observed in the present study.

There are limitations in this study. First, the difficulty in following patients because only 13 of 23 continued under our supervision. Second, in some patients we have short follow up including cases with only 14 months which turned impossible to do disease-free survival analysis, for example. Nevertheless, we brought a significant number of cases accompanied by long years with a significant success rate using the modified carnoy’s solution, showing that this can be an alternative in surgical management.

## Conclusions

Thus, it can be concluded that the OM exhibits a well-defined clinical profile, occurring predominantly in patients of the second and third decades of life, in the posterior mandible region and that the choice of treatment should consider aspects such as the patient’s age, size of the injury and location.

The conservative approach was an effective therapy with lower morbidity and not associated with high recurrence rates. Furthermore, dental surgeons should be aware of tumor recurrence and keep a strict follow-up protocol.
